# Fish diversity in the middle and lower reaches of the Ganjiang River of China: Threats and conservation

**DOI:** 10.1371/journal.pone.0205116

**Published:** 2018-11-02

**Authors:** Qin Guo, Xiongjun Liu, Xuefu Ao, Jiajun Qin, Xiaoping Wu, Shan Ouyang

**Affiliations:** 1 School of Life Sciences, Nanchang University, Nanchang, China; 2 Poyang Lake Key Laboratory of Environment and Resource Utilization (Nanchang University), Ministry of Education, Nanchang, China; 3 School of Resource, Environment and Chemical Engineering, Nanchang University, Nanchang, China; 4 Center for Watershed Ecology, Institute of Life Science, Nanchang University, Nanchang, China; Pontificia Universidade Catolica do Rio Grande do Sul, BRAZIL

## Abstract

The Ganjiang River has abundant fish resources, which plays a significant role in maintaining and replenishing the fish resources in Poyang Lake and the Yangtze River, and contains important habitat for migratory fish. However, fish diversity has rapidly declined in the Ganjiang River, especially migratory fish. In this study, 107 fish species (including 43 Chinese endemic species) were found in the middle and lower reaches of the Ganjiang River. However, only 91 fish species were found in the main channel of the Ganjiang River, which was lower than the 108 fish species historically found there. According to the Chinese Red List, 85 Least Concern, two Critically Endangered, three Vulnerable, one Near Threatened and 16 Data Deficient fish species were found in the Ganjiang River. In addition, the species number, diversity and CPUE in the channel were all greater than in the reservoir. The Bray-Curtis resemblance matrix and non-metric multidimensional scaling (NMDS) showed that the habitats of the Ganjiang River were divided into three areas. The analysis of RDA showed that turbidity, dissolved oxygen and water depth significantly affected fish distributions and assemblage composition. These results indicated that dam construction and other human activities have seriously destroyed the fish habitat and led to the decline in fish diversity. Therefore, the conservation of fish has become urgent in the Ganjiang River, and an integrated management plan should be developed and effectively implemented.

## Introduction

Freshwater fish are not only the most diverse group of vertebrates but also have the greatest proportion of threatened species [1–4]. Fish assemblages are also an important element in aquatic ecosystems, which are used as one of four biological indicators for aquatic ecosystem assessment [5–8]. However, freshwater fish had reduced ability for inter-basin movement in the relatively limited space [7, 9], in contrast marine fish had the relatively free movement in the broad space, which was at the root of the conservation problems of the former [3, 10]. In addition, fish are important elements of the economy for many nations as they have been a staple to the diet of many people. Over the past few decades, fish resources decreased dramatically, and endemic species have faced continuous threats globally. Dams, overfishing, pollution, deforestation, land erosion and other human activities are considered as the main threats to fish biodiversity [3, 11–12]. Therefore, the conservation of fish biodiversity has become more important.

The Ganjiang River is the largest river running from north to south in Jiangxi Province, China, flowing into Poyang Lake and is the seventh largest tributary of the Yangtze River, and has abundant fish resources and important habitat for migratory fish [4]. Therefore, the Ganjiang River plays a significant role in maintaining and replenishing the fish resources in Poyang Lake and the Yangtze River [4]. However, many dams have been constructed in the Ganjiang River, such as the Wanan Dam, Shihutang Dam and Xiangjiang Dam [4]. Four spawning areas have disappeared from the Ganjiang River, and the population of *Tenualosa reevesii* was almost extinct after construction of the Wanan Dam in 1990 [4, 13–14]. Dam construction has been shown to have a profound effect on watersheds and aquatic ecosystems [3–4, 15]. Many studies [4, 16–17] have shown that fishes are the most sensitive organisms affected by dam construction and flow regulation. Specifically, migratory fish are affected the most seriously, because accesses to spawning grounds are disrupted by dams since all fish (migratory or otherwise) have disrupted longitudinal connections [18–20]. Dams also caused habitat fragmentation and loss [17, 20, 21], leading to the loss of native fish species, the invasion of exotic fish species [22], a decline in fish beta-diversity and a resulting increase in faunal similarity or biotic homogenization [22–23]. In addition, overfishing, pollution, sand extraction and other human activities have seriously destroyed fish habitats and led to a decline in fish diversity [3–4, 7, 12]. Since the 1950s, fish populations have been declining and population structures have decreased and become younger. In addition, the amount of endangered species has increased and some species are nearing extinction (such as *Acipenser sinensis*, *Luciobrama macrocephalus*, *Tenualosa reevesii*, *Ochetobius elongatus* and *Myxocyprinus asiaticus*), which immediately threatened fish diversity in the Ganjiang River [24]. Despite many studies have investigated fish resources in some areas of the Ganjiang River, no comprehensive study has been conducted on fish diversity and conservation status, nor has there been consideration of species diversity and the ecological characteristics of the habitat. The objective of this study was to analyze the species composition and diversity of the fish fauna in the middle and lower reaches of the Ganjiang River, and explore the correlation of environmental factors and fish diversity. For that, two hypotheses were tested: (1) fish composition and diversity in the middle and lower reaches of the Ganjiang River have declined; (2) changes of turbidity, dissolved oxygen and water depth would affect fish distributions and assemblage composition.

## Materials and methods

### Ethics statement

The study was approved by the Institutional Animal Care and Use Committee (IACUC) of Nanchang University, Jiangxi, China. All necessary permits were obtained for the described field studies from the IACUC of Nanchang University and the Yangtze River Fishery Administration of China. The handling of fish was also conducted in accordance with the guidelines on the care and use of animals for scientific purposes set by IACUC of Nanchang University, Jiangxi, China.

### Study area

The Ganjiang River (116°01′- 116°22′ E, 25°57′ - 29°11′ N) is the largest river in Jiangxi Province, China. It covers a total catchment area of 82809 km^2^, with a main channel of 766 km. In addition, it is a complex river system and belongs to the middle-subtropical humid monsoon climate zone. Precipitation is abundant with an average of 1580.8 mm/year. The upper reaches of the river are above of the Ganzhou city (255 km), a mountainous streams of the river, with many streams and abundant water resources; the middle reaches of the river is from the Ganzhou city to the Xingan county (303km) which water flow is generally smooth, and the river bed is mostly coarse, fine sand and red gravel; and the lower reaches of the river is from the Xingan county to the Wucheng town which is 208 km long, and finally flow into Poyang Lake at the Wucheng town.

### Sampling sites

**Fig 1:**
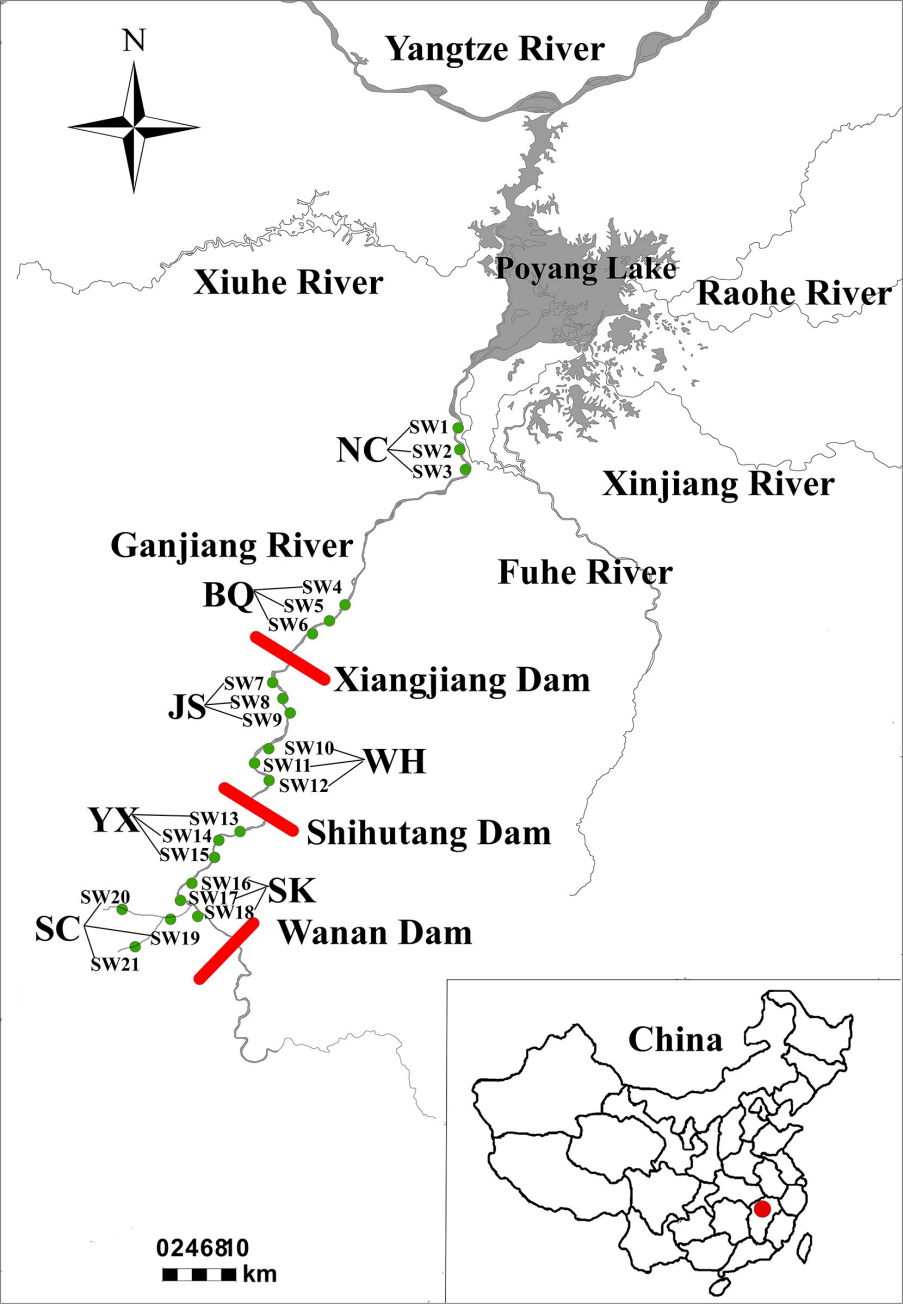
Sampling sections locations in the middle and lower reaches of the Ganjiang River. NC: Nanchang; BQ: Baqiu; JS: Jiangsha; WH: Wanhe; YX: Yanxi; SK: Shukou; and SC: Suichuan.

In the study, sampling sites was selected by considering habitats variation and anthropogenic activities in the middle and lower reaches of the Ganjiang River. We established seven sections (21 sampling sites) in the middle and lower reaches of the Ganjiang River (Fig 1), and each section was subdivided into three sampling sites that included (1) channel: Nanchang (Section code was NC; SW1, SW2 and SW3), Baqiu (Section code was BQ; SW4, SW5 and SW6), Jiangsha (Section code was JS; SW7, SW8 and SW9), which had abundant vegetation, rapid water flow and riverbed with sandstone, sandy substrate and gravel; (2) reservoir: Wanhe (Section code was WH; SW10, SW11 and SW12), Yanxi (Section code was YX; SW13, SW14 and SW15), Shukou (Section code was SK; SW16, SW17 and SW18), which had slow water flow and riverbed with sandy substrate; and (3) tributary: Suichuan (Section code was SC; SW19, SW20 and SW21), which had abundant vegetation, shallow water, rapid water flow and riverbed with sandstone, rock and gravel (S1 Table). Fish samples were collected from December (dry season) 2015 to July (wet season) 2016.

### Sampling methods

In this study, professional fishermen were hired to capture fish with multiple mesh-sized gillnets in the middle and lower reaches of the Ganjiang River. At each site, five sampling points were selected with similar average depths, and we assumed similar capture efficiencies from gillnet samples at each site. Sampling was fully standardized using five gillnet clusters, each consisting of six gillnets of 50–80 m in length 4–10 m in height (mesh size = 1.0–10.0 cm), amounting to a total sampling area of 625 m^2^. Nets were exposed for 12 h (21:00 h to 09:00 h the following day) and were emptied every 4 h. Additional collections using alternative fishing techniques (cast nets, long-lines and small mesh gill nets) were also performed during standardized sampling times to enhance the species checklists at each section. Fish species were counted and weighed in the field, and the unidentified species were fixed in a 10% formaldehyde solution and further identified in the laboratory. Live fish were released at the sampling sites. At the same time, we surveyed and collected fish in the township markets along the river to enhance the species checklists at each section. All fish specimens were identified according to Chen (1998), Chu et al. (1999), and Yue (2000) [25–27], and the scientific name was corrected according to Fishbase (http://www.fishbase.org/search.php). The division of the ecological fish types was according to the Institute of Hydrobiology, Chinese Academy of Sciences [28]. Life habits were divided into eurytopic, rheophilic, limnophilic and diadromous; feeding habits were divided into piscivores, zoobenthivores, insectivores, omnivores, zooplanktivores, phytoplanktivores, phytobenthivores and herbivores; water layer habitats were divided into upper layer, lower layer, and demersal [28]. The abundance of each species was expressed in terms of catch per unit effort (CPUE; number of fish per hour, unit: ind / h).

Environmental factors were measured at each location for each sampling site. Dissolved oxygen (DO), hydrogen ions (pH), turbidity (Turb), total dissolved solids (TDS) and salinity (Sal) were measured using a YSI 650MDS (USA) multiparamenter meter; water velocity, water depth and chlorophyll-a (Chl-a) were measured with a global water velocity meter (FP111), digital sonar (H22px handheld sonar system) and chlorophyll meter (HL-168C06, made in China), respectively.

### Data analysis

We assessed the sampling completeness of fish for each sampling section using abundance-based rarefaction as implemented in iNext online [29]. Confidence intervals (95%) were calculated using100 bootstrap replications.

The index of relative importance (*IRI*) based on number percentage, weight percentage, and frequency of occurrence was used to measure fish dominance in catches and calculated as follows:

$$IRI_{i}=\left(\%N_{i}+\%W_{i}\right)\times f_{i},$$ (1)

where %*N*_*i*_ and %*W*_*i*_ were percentage number and percentage weight, respectively, of species *i* in the total catches, and %*f*_*i*_ was the occurrence frequency of species *i* [30]. An IRI >10% indicated the fish species was dominant [30].

The relative abundance of each species at each sampling site was estimated by:

$$P_{i}=N_{i}/\sum_{j=1}^{s}N_{j}$$ (2)

where *S* = number of species and *N*_*i*_ and *N*_*j*_ were the counts of individual species in the sample. The Shannon-Wiener index (*H'*: *H'* = –∑*P*_***i***_ln*P*_***i***_), Simpsonʹs Index (*D*_*s*_: *D*_*s*_ *=* 1–∑(*P*_*i*_)^2^), and Pielou evenness index (*J'*: *J'* = *H'*/ln*S*) were used to calculate fish species richness for each section [31–32], where *S* = the total number of species in each sample collected in the river.

To analyze the habitat differences in species abundance in the seven sections (21 sampling sites), we performed a Bray-Curtis resemblance matrix based on the fish species per section. The data were square-root transformed to reduce the effect of the highly abundant species. The resemblance matrix was used to create a two-dimensional, non-metric multidimensional scaling (NMDS) plot [33]. Additionally, similarities were analyzed by a hierarchical cluster analysis using group means. We used ANOSIM tests to assess assemblage similarity between sampling sites and seasons. SIMPER tests were used to determine the contributions of each species to any differences. These operations were performed with the software Primer 6 [34]. Bar charts and their metrics were calculated using R [35].

A redundancy analysis (RDA), which was a multivariate direct gradient analysis technique, was used to evaluate the variations of species composition in correlation with environmental variables [4, 36]. The correlations between fish species composition and the measured environmental factors were analyzed by RDA [4, 36]. Detrended correspondence analysis indicated that our aquatic dataset had a short gradient length, suggesting that the linear model of RDA was more appropriate than a canonical correspondence analysis [36]. All variables were entered in the analysis after a forward selection procedure to show their importance in explaining the total variability in the species composition [36]. The significance (*p* < 0.05) of the RDA gradient was assessed by Monte Carlo permutation tests, and their importance was measured by the eigenvalues of the first two axes [36]. All species composition and environmental data were log10(X+1) transformed to meet the assumptions of multivariate normality and to moderate the influence of extreme data. All the ordinations were performed using CANOCO 4.5 [36].

## Results

### Fish species composition

19355 fish specimens were sampled in the middle and lower reaches of the Ganjiang River that were identified into 107 species, 69 genera and 18 families (S2 Table). The number of Cypriniformes was the greatest, accounted for 67.3% of the total, followed by Siluriformes, accounting for 15.0%, Beloniformes and Synbranchiformes was the lowest, accounted for 0.9% each. The dominant species were *Squalidus argentatus*, *Cyprinus carpio*, *Carassius auratus* and *Silurus asotus* (S3 Table). Additionally, there were 43 Chinese endemic species (S4 Table), which accounted for 40.2% of the total. Moreover, according to the Chinese Red List [37], Least Concern fish species were the greatest, which accounted for 79.4% of the total (S4 Table). Critically Endangered fish species were *Ochetobius elongatus* and *Myxocyprinus asiaticus*; Vulnerable fish species were *Leptobotia elongata*, *Pseudobagrus pratti* and *Siniperca roulei*; and Near Threatened fish species was *Siniperca obscura* (S4 Table). Sampling completeness was relatively high, with Chao I measures estimating more than 95% of each sampling section. The final slopes of the observed and estimated species accumulation curves for fish at each section were closed to asymptotic (S1 Fig).

The analysis of feeding habits showed that insectivores were dominant, accounting for 33.6% of the total, followed by carnivorous (27.1%), and phytoplanktivorous (1.9%; S4 Table). The habitat characteristics analysis indicated that the number of demersal were the greatest, accounting for 46.7% of the total, followed by lower-layer (33.6%) and upper-layer (19.7%; S4 Table). The comparison of fish life habits revealed that the number of eurytopic were the greatest, accounting for 45.8% of the total, followed by rheophilic (38.3%) and river-lake migration fish species (6.5%; S4 Table).

### Fish assemblage structure

The diversity of fish in the sampling sections was calculated from the survey data (Table 1). The results showed that the diversity of Suichuan (SC) was the greatest (*H′* = 3.24, *J'* = 0.77, *D*_s_ = 0.93). In addition, the diversity of the tributary was the greatest, followed by the channel, and the reservoir was the lowest. Fish diversity in the wet season was greater than the dry season.

**Table 1:** Diversity index of fish assemblages among the seven sections in the middle and lower reaches of the Ganjiang River.

Sampling sites	Species number	Shannon- Wiener index (*H′*)	Simpson index (*D*_s_)	Pielou index (*J'*)
NC	66	2.9	0.91	0.69
BQ	67	3.11	0.93	0.74
JS	38	2.45	0.86	0.67
SHT	41	2.34	0.76	0.63
YXD	42	2.71	0.86	0.73
SK	34	2.83	0.9	0.8
SC	68	3.24	0.93	0.77

**Fig 2:**
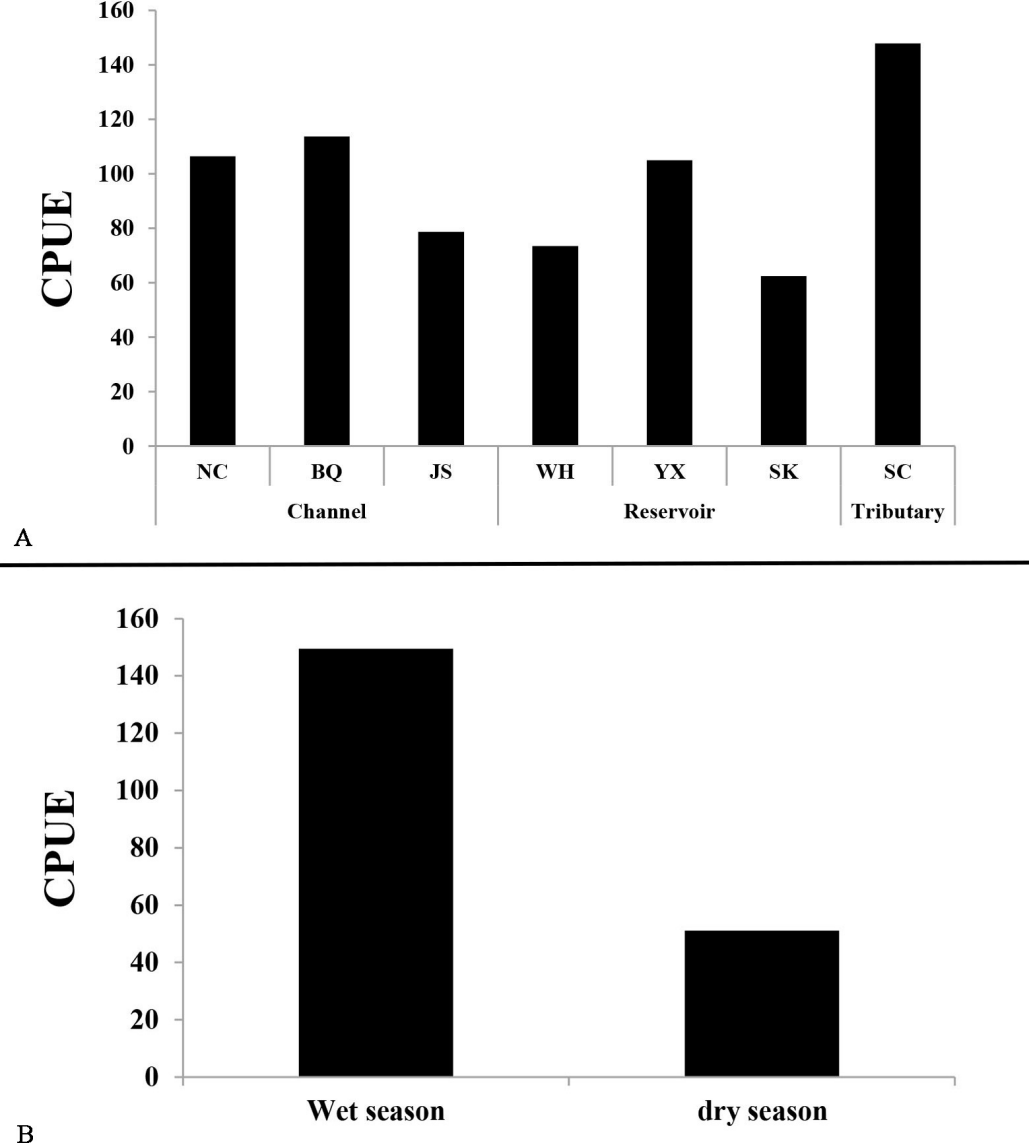
Comparison of CPUE for fish composition across sections (A) and sampling periods (B) in the middle and lower reaches of the Ganjiang River.

The abundance of each species was expressed in terms of catch per unit effort (CPUE) in the middle and lower reaches of the Ganjiang River. The results showed that the CPUE in the tributary was the greatest (147.79), followed by the channel, and the reservoir was the lowest (Fig 2A). In addition, the CPUE in the wet season was greater than the CPUE in the dry season (Fig 2B).

**Fig 3:**
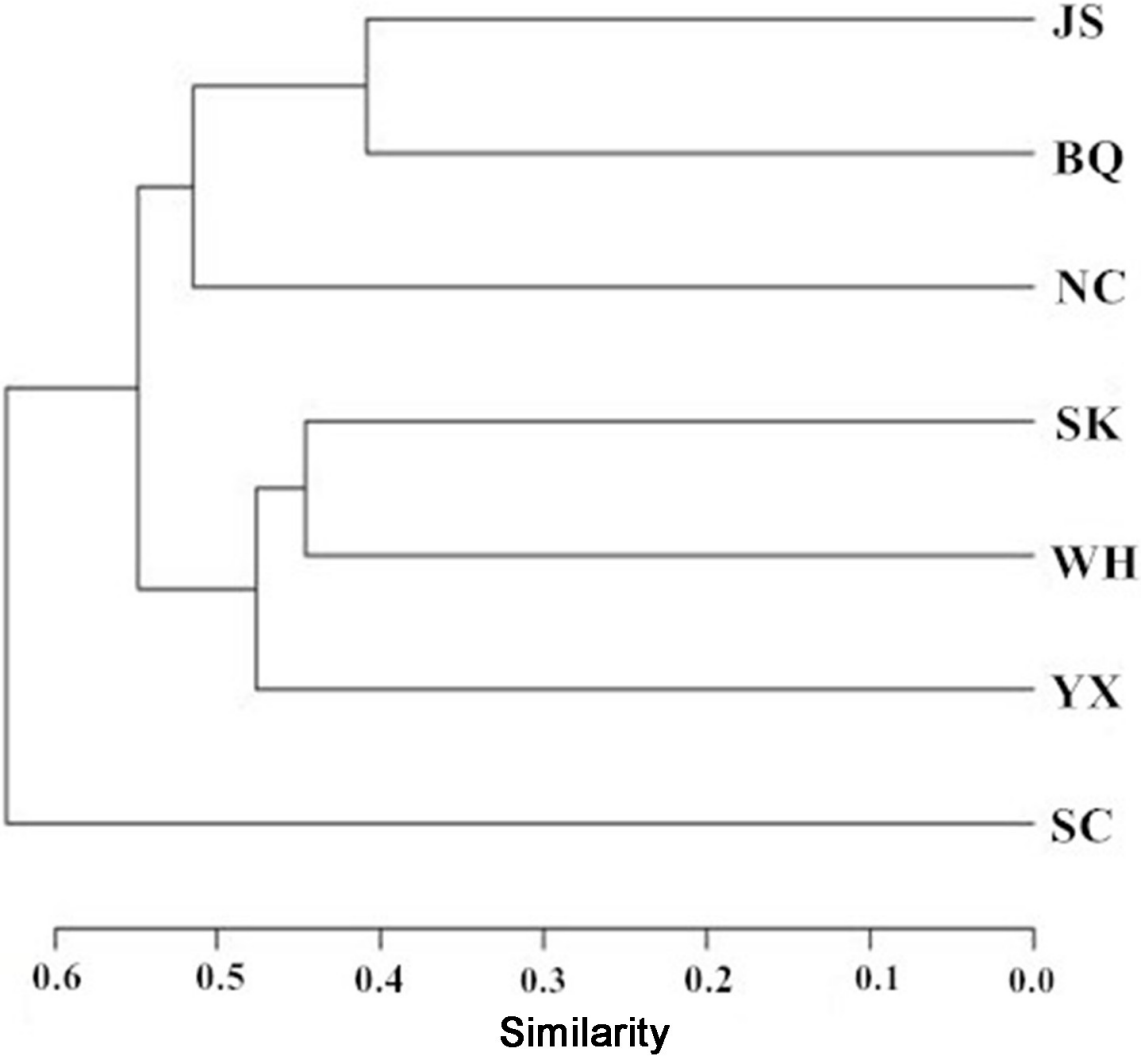
The classification in the middle and lower reaches of the Ganjiang River using the Bray-Curtis resemblance matrix. NC: Nanchang; BQ: Baqiu; JS: Jiangsha; WH: Wanhe; YX: Yanxi; SK: Shukou; and SC: Suichuan.

**Fig 4:**
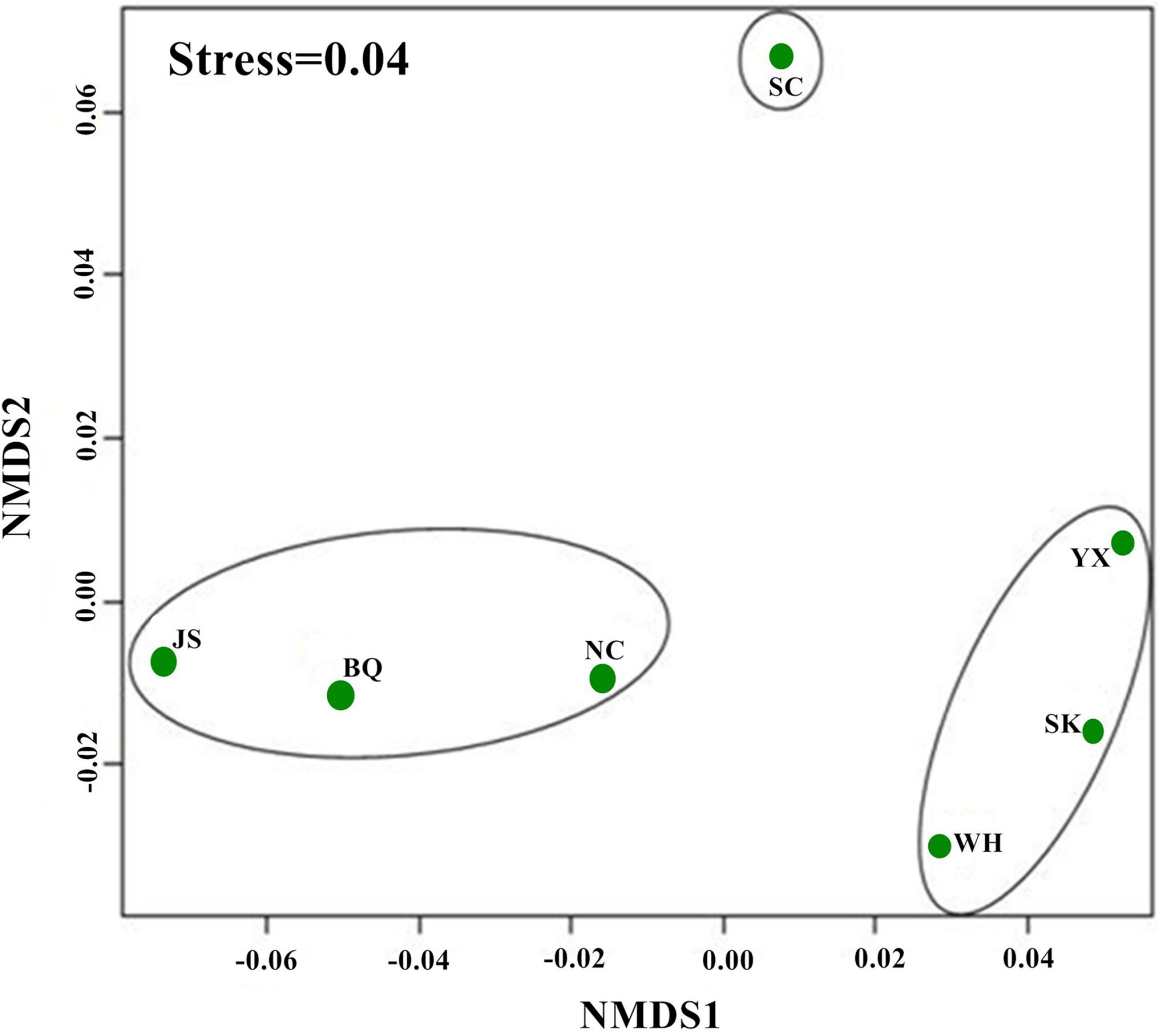
NMDS ordination of the fish community in the middle and lower reaches of the Ganjiang River. NC: Nanchang; BQ: Baqiu; JS: Jiangsha; WH: Wanhe; YX: Yanxi; SK: Shukou; and SC: Suichuan.

The Bray-Curtis resemblance matrix showed that the middle and lower reaches of the Ganjiang River were divided into three areas (Fig 3). The first area included Nanchang section (NC), Baiqiu section (BQ) and Jishui section (JS), which were the fish assemblage structures of the channel; the second area included Wanhe section (WH), Yanxi section (YX) and Shukou section (SK), which were fish assemblage structures of the reservoir; the third area included Suichuan section (SC), which was the fish assemblage structure of the tributary. The results of the NMDS was coincident with the Bray-Curtis resemblance matrix, indicating the results were reliable (stress = 0.04; Fig 4). The analysis of ANOSM (*R* = 0.0778; *P* = 0.013) showed that habitat difference among the three areas of the river was significant (*R<*1; *P<*0.05).

The dissimilarity analysis (SIMPER) was conducted between fish assemblages sampled at the three areas in the middle and lower reaches of the Ganjiang River. The 21 species that provided the greatest contribution are shown for each section-pair comparison. The results of the fish assemblage structure channel/reservoir showed that the greatest contribution was *Chanodichthys dabryi*, the greatest contribution to the channel/tributary was *Gobiobotia filifer*, and the greatest contribution to the tributary/reservoir was *Carassius auratus* (Table 2).

**Table 2:** Dissimilarity analysis (SIMPER) between fish assemblages in the middle and lower reaches of the Ganjiang River. The 21 species provided the greatest contribution and were shown for each site-pair comparison. NS: Community differences were not significant.

Species	Channel / Reservoir	Channel / Tributary	Tributary / Reservoir
*Squalidus argentatus*	13.86%	27.37%	30.43%
*Rhinogobius giurinus*	21.81%	51.78%	NS
*Tachysurus fulvidraco*	29.53%	58.59%	50.28%
*Pseudobrama simoni*	37.16%	55.51%	46.15%
*Hemiculter bleekeri*	44.56%	43.27%	NS
*Saurogobio dabryi*	50.55%	NS	41.59%
*Pseudorasbora parva*	54.40%	NS	57.01%
*Carassius auratus*	58.10%	47.55%	71.24%
*Hemiculter leucisculus*	61.07%	NS	NS
*Gobiobotia filifer*	63.74%	70.99%	NS
*Pelteobagrus eupogon*	65.91%	NS	NS
*Tachysurus nitidus*	68.03%	NS	NS
*Chanodichthys dabryi*	70.12%	NS	NS
*Pseudohemiculter dispar*	NS	14.82%	17.18%
*Acrossocheilus parallens*	NS	33.55%	24.56%
*Zacco platypus*	NS	38.83%	36.27%
*Vanmanenia stenosoma*	NS	61.56%	53.82%
*Glyptothorax sinensis *	NS	64.23%	60.20%
*Opsariichthys bidens*	NS	66.59%	66.42%
*Abbottina rivularis*	NS	68.88%	63.34%
*Leptobotia elongata*	NS	NS	68.85%

### The correlation of environmental factors and fish composition

**Fig 5:**
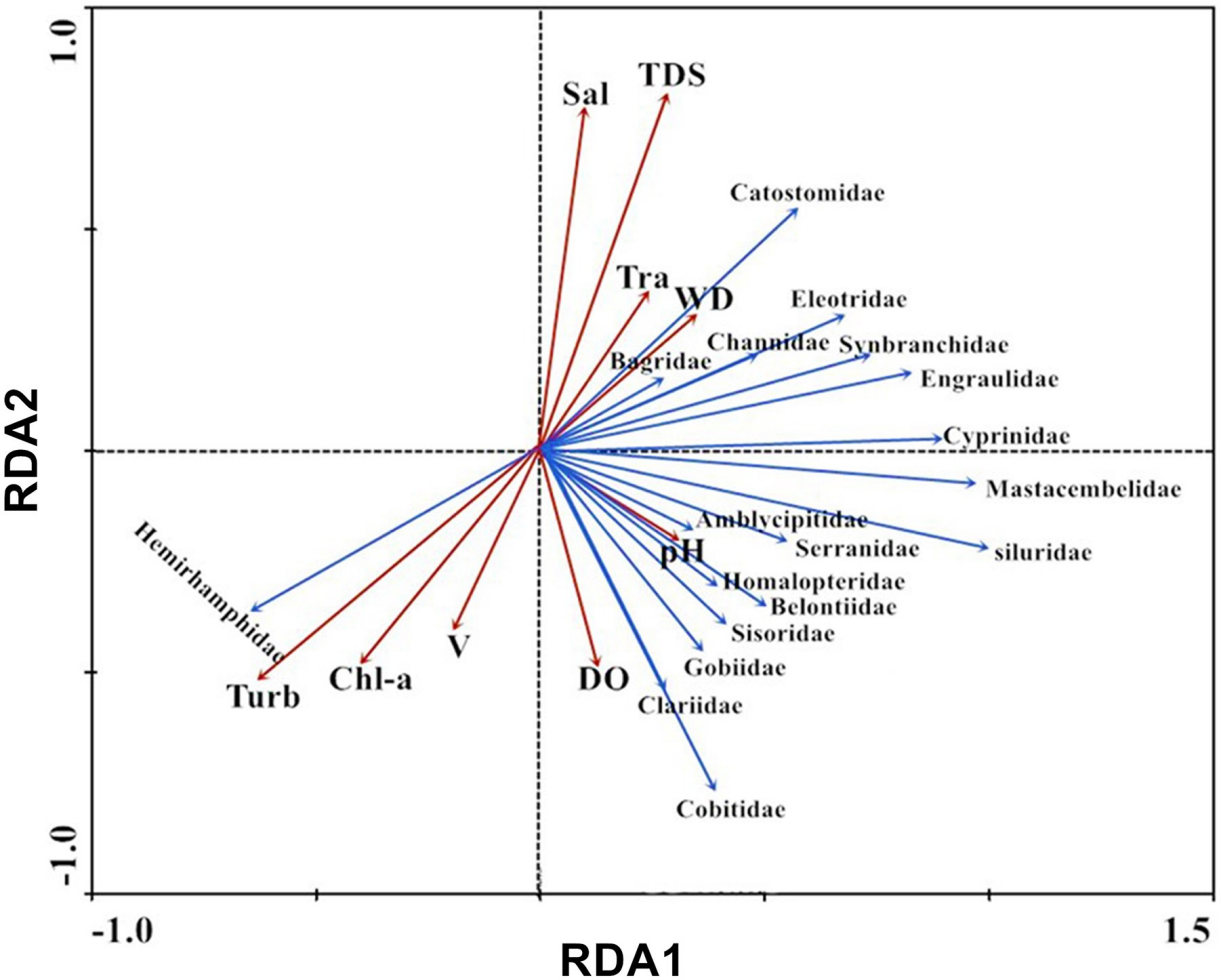
Ordination biplot of fish species assemblages and environmental variables obtained by RDA across sampling periods and sites.

The analysis of environmental factors showed that pH, DO, salinity, turbidity, water depth, water velocity and Chl-a ranged from 6.26 to 7.58, 7.05 to 12.35 mg/L, 0.02 to 0.06 mg/L, 3.40 to 16.95 NTU, 1.30 to 16.90 m, 0.10 to 1.50 m/s and 1.1to 3.12 μg/L, respectively (Table 3). Biplots were generated using RDA after extracting and integrating data from the fish community indices with the 8-physicochemical parameter (pH, turbidity, DO, water depth, water velocity, salinity, TDS, Chl-*a* and transparency) data matrix (Fig 5). The results showed that the first axis of eigenvalues was 0.903, indicating that the environmental gradient was broadly applied to the ordination analysis. The first axis of the cumulative percentage of variance of the species–environmental relation was 90.3%, and the four axes made up 98.7%. Catostomidae, Bagridae, Channidae, Eleotridae, Synbranchidae, Engraulidae and Cyprinidae were correlated with pH, salinity, water depth, transparency and TDS; Mastacembelidae, Siluridae, Amblycipitidae, Serranidae, Homalopteridae, Belontiidae, Sisoridae, Gobiidae, Clariidae and Cobitidae were correlated with pH, DO and water velocity. Hemirhamphidae were correlated with water velocity, turbidity and Chl-*a* (Fig 5). In general, turbidity, dissolved oxygen and water depth significantly affected fish distributions and assemblage composition (*P*<0.05).

**Table 3:** Environmental factor changes in the middle and lower reaches of the Ganjiang River.

	NC		BQ		JS		WH		YX		SK		SC	
	Mean	SD	Mean	SD	Mean	SD	Mean	SD	Mean	SD	Mean	SD	Mean	SD
pH	7.50	0.08	6.30	0.04	6.60	0.06	7.21	0.02	7.30	0.04	7.20	0.08	7.39	0.06
Turb (NTU)	10.70	0.15	11.70	0.15	13.50	0.26	10.80	0.25	17.50	0.19	16.80	0.15	11.98	0.25
Chl-a (μg/L)	1.30	0.21	1.60	0.15	1.70	0.21	1.50	0.12	1.80	0.05	3.00	0.12	2.30	0.05
DO (mg/L)	9.28	1.28	9.18	2.13	9.30	1.32	9.15	1.31	9.40	2.95	9.30	0.56	9.45	1.57
Sal (mg/L)	0.05	0	0.05	0.01	0.05	0	0.04	0.05	0.02	0	0.04	0.01	0.02	0
TDS (μs/cm)	73.5	16.3	70.0	11.6	75.0	8.5	71.0	9.1	42.0	6.1	37.0	6.8	49.0	8.4
Tra (m)	0.60	0.12	1.10	0.13	0.90	0.09	0.80	0.10	0.50	0.21	0.60	0.04	0.80	0.07
WD (m)	8.0	1.2	12.6	2.6	9.0	0.9	2.5	0.8	5.6	0.9	2.4	0.4	2.0	0.5
V (m/s)	0.20	0.05	0.20	0.02	0.30	0.02	0.60	0.03	0.30	0.01	0.80	0.01	0.90	0.01

## Discussion

### Fish biodiversity and its threat factors

Fishes are the most studied group of species and the best indicators of geographical patterns [38]. Due to high levels of endemism and human pressure, freshwater fish fauna around the world are under serious threat [39]. Threats to freshwater fish species require special attention because historical influences on the distribution and diversity patterns may be more evident in freshwater fishes than in other taxonomic groups, and detailed patterns of endemism and distribution of freshwater fishes differ from those in birds and mammals [24, 40]. In this study, 107 fish species were found in the middle and lower reaches of the Ganjiang River. However, except for 68 fish species in the tributary, only 91 fish species occurred in the main channel of the Ganjiang River. Historically, 176 and 108 fish species were found in the main stem of the Ganjiang River, in 1982–1991 and 2007–2010, respectively (S5 Table) [41–46]. In addition, six fish species had seriously declined such as *Acipenser sinensis*, *Luciobrama macrocephalus*, *Tenualosa reevesii*, *Ochetobius elongates*, *Myxocyprinus asiaticus* and *Coilia nasus*, and *Acipenser sinensis*, *Luciobrama macrocephalus* and *Tenualosa reevesii* may even be extinct. The results of species composition indicated that fish diversity in the Ganjiang River was rapidly decreasing [4, 47].

Dam construction has been shown to have a profound effect on aquatic ecosystems, and caused habitat fragmentation and loss [4, 17, 20–21, 48]. Historically, Poyang Lake was interwoven with the Yangtze River and the mainstream and tributaries of five rivers (Ganjiang River, Xinjiang River, Raohe River, Xiuhe River and Fuhe River) to form a complex lacustrine–riverine network [47]. Fish could move freely in this system, from one river to another, through or across the large lake, and among the tributaries. Wanan Dam was constructed in the Ganjiang River, resulting in the disappearance of four spawning areas in 1991[14]. However, dams still were constructed in the Ganjiang River, such as the Shihutang Dam, Xiangjiang Dam, Xingan Dam, and Longtoushan Dam [4]. Specifically, Xiangjiang County is very important spawning areas (important migratory fish spawning areas) in China. The Xiangjiang Dam has been constructed in the Ganjiang River, resulted in some fish species disappeared and the decline of fish resources. Currently, only the lower reach of the Ganjiang River can still connect to Poyang Lake and the Yangtze River. However, dam constructions in the lower reach of Ganjiang River and Poyang Lake will cut off the connectivity among the Ganjiang River, Poyang Lake and the Yangtze River in the future. In this study, the results of the Bray-Curtis resemblance matrix and NMDS showed that the habitats of the Ganjiang River were divided into three areas, including the channel, reservoir and tributary.

Many studies have shown that fish assemblages were also the most sensitive organisms to dam construction [4, 17, 49–50]. Migratory fish are especially affected when longitudinal connections are disrupted by dams [19–20, 48], because migratory fish require upriver movements toward spawning grounds at the beginning of the rainy season; eggs then drift downriver while they develop and hatch in the turbid waters at the beginning of the flooding period [17, 51]. For example, the spawning migrations of *Acipenser sinensis* were blocked by the Gezhouba Dam, resulting in a great decline of this relatively abundant species [52]; The Three Gorges Dam (the first dam) drastically changed the hydrology of the Yangtze River and negatively affected the survival of fish species [49–50]. In this study, fish species abundance in the channel was the greatest, containing 91 species, followed by the tributary (68), and the reservoir had the lowest species abundance (60). In addition, the CPUE and diversity in the channel were both greater than in the reservoir. Migratory fish resources in the channel were greater than in the reservoir. The results indicated that dams fragmented and disrupted the natural flow regime of over half of the large river systems and threaten the survival of fish [4, 17, 53].

The loss of habitat from drainage has contributed to fish declines in the Ganjiang River. The Ganjiang River covered a total area of approximately 82809 km^2^. However, some river area was drained for farming, and 16709 km^2^ of water area disappeared [54–55]. Therefore, the loss of river area was one of the most important factors affecting aquatic biodiversity. Overfishing was also considered a key reason for the decline of fisheries [56]. For example, the yield of *Tenualosa reevesii* rapidly declined from 309–584 t in 1960, from 74–157 t in 1970, and 12 t in 1986 due to overfishing [57]. Sand extraction was also a serious problem in the Ganjiang River because it could damage the habitat required by fish for feeding, migration and reproduction [55, 58–59]. For example, the turbidity of the water has increased 50 times from 1998 to 2004 due to sand excavation, resulting in a 0.3 km^2^ grass island that slid into Poyang Lake in 2004 [60]. Water pollution was also a serious problem in the Ganjiang River and affected the survival of fish. For example, due to discharges from the Dexing copper mines and other mines along the Jishui River, heavy metal water pollution has affected the aquatic ecosystem [61].

### Effects of physicochemical parameters on fish composition and biodiversity

River longitudinal connectivity and hydrological conditions significantly affected the aquatic community structure in the river ecosystem [4, 17]. The biotic composition, structure and aquatic ecosystem function were determined by the hydrologic regime, and it affected aquatic biodiversity via several interrelated mechanisms operating over different spatial and temporal scales [4, 15–17, 20]. River discontinuity caused by dam construction resulted in significantly changed hydrological conditions, thereby influencing the aquatic community structure [4]. Due to water dynamic changes and the effects of barriers, dam construction and other human activity limited the flow of nutrients, organisms, matter, energy and genetic information in aquatic habitats [20–21, 62]. Many studies have also found that the changes of environmental factors, such as dissolved oxygen and pH [63], water depth [64], current velocity [65], and turbidity [66] affected fish assemblages. In this study, the analysis of RDA showed that six families were correlated with pH, salinity, water depth, transparency and TDS; ten families were correlated with pH, DO and water velocity; and one family was correlated with water velocity, turbidity and Chl-a. In general, turbidity, dissolved oxygen and water depth significantly affected fish distributions and assemblage composition (*P*<0.05).The results indicated that environmental factors also affected fish distributions and assemblage composition [67].

### Conservation implications of fish biodiversity

The Ganjiang River was experiencing very rapid habitat fragmentation due to human disturbance and their subsequent environmental change, and conservation strategies must be improved and expanded [12]. Currently, conservation of fish biodiversity mainly focused on endangered and economic fishes [12, 68]. It has taken a variety of measures to protect fish diversity in China and even in global [12, 20], but we believed that these efforts were still inadequate. To restore fish communities in the river, the following measures should be implemented: First, the habitats rich in endemic species should be identified as nature reserves [69]. For example, the Ganjiang River had complex habitats, abundant fish resources and endemic species. However, no effective actions have been taken to mitigate the possible decline of fish diversity. In addition, approximately 156 reserve areas for the conservation of plants, animals and wetlands have been established in Jiangxi Province, but there are no freshwater protected areas nor are there any fish passage facilities in the rivers of Jiangxi Province [47]. Therefore, it should set up more nature reserves in important freshwater areas. Second, it should strengthen conservation of migratory fish habitats. Conservation strategies must carefully consider the complex life cycles of migratory fish species and their ontogenetic shifts in habitat. Dam construction fragments habitats resulting in obstruction of migration paths for fish. Therefore, dam construction should be sited after careful consideration of the life histories of the ichthyofauna so that their impacts on migratory patterns can be mitigated. Third, study on the life histories of fish species should be strengthened, especially threatened fish species. In this study, six threatened fish species need to be given attention, because explicit information on the life histories of threatened fish was highly necessary for conserving fish diversity. Fourth, fishery sanctuaries need to be established. Abundant fish resources and complex habitats in the Suichuan River may be treated as a type of sanctuary. Fifth, closed fishing seasons need to be ordered. A large number of fishing methods such as traps, gill nets, and electrofishing are employed, resulting in overfishing, which has also caused a dramatic decline in fish diversity [45, 58].

## Conclusions

The Ganjiang River plays a significant role in maintaining and replenishing the fish resources in Poyang Lake and the Yangtze River [4]. However, fish resources have rapidly declined in the Ganjiang River [4]. The results have further shown that fish composition and diversity has rapidly declined in the Ganjiang River. The analysis of the Bray-Curtis resemblance matrix and the NMDS showed that the habitats of the middle and lower reaches of the Ganjiang River were divided into three sections. The analysis of RDA showed that turbidity, dissolved oxygen and water depth significantly affected fish distributions and assemblage composition. These results indicated that anthropogenic activities and their subsequent environmental disturbances may have seriously destroyed the fish habitat and led to the decline of fish diversity. At the same time, it would have also potential impacts on fish diversity of Poyang Lake and the Yangtze River. Thus, the conservation of fishes has become urgent, and an integrated management plan should be developed and effectively implemented.

## Supporting information

S1 Fig Species accumulation curves for fish at each sampling section in the middle and lower reaches of the Ganjiang River. Shaded areas represent the 95% confidence intervals.
(PDF)

S1 Table Habitat characteristics of the seven sections in the middle and lower reaches of the Ganjiang River. (DOCX)

S2 Table Fish composition and distribution in the middle and lower reaches of the Ganjiang River. (DOCX)

S3 Table Comparison of fish composition by sample period and sampling sections in the middle and lower reaches of the Ganjiang River. (DOCX)

S4 Table Fish ecotype and Chinese Red List [37] in the middle and lower reaches of the Ganjiang River. Eu: Eurytopic; R: Rheophilic; L: Limnophilic; D: Diadromous; C: Piscivores; Zb: Zoobenthivores, I: Insectivores; O: Omnivores; Z: Zooplanktivores; P: Phytoplanktivores; Pb: Phytobenthivores; Herbivores; UL: Upper layer; LL: lower layer; De: demersal. △: Chinese endemic species.
(DOCX)

S5 Table Historical fish composition and distribution in the middle and lower reaches of the Ganjiang River. (DOCX)
