# Supplementary material for: Fish diversity in the middle and lower reaches of the Ganjiang River of China: Threats and conservation
Source: PLoS One. 2018 Nov 2;13(11):e0205116. doi: 10.1371/journal.pone.0205116 (PMC6214499; doi:10.1371/journal.pone.0205116)
Supplement: S1 Fig — Shaded areas represent the 95% confidence intervals. (PDF) [file pone.0205116.s001.pdf]

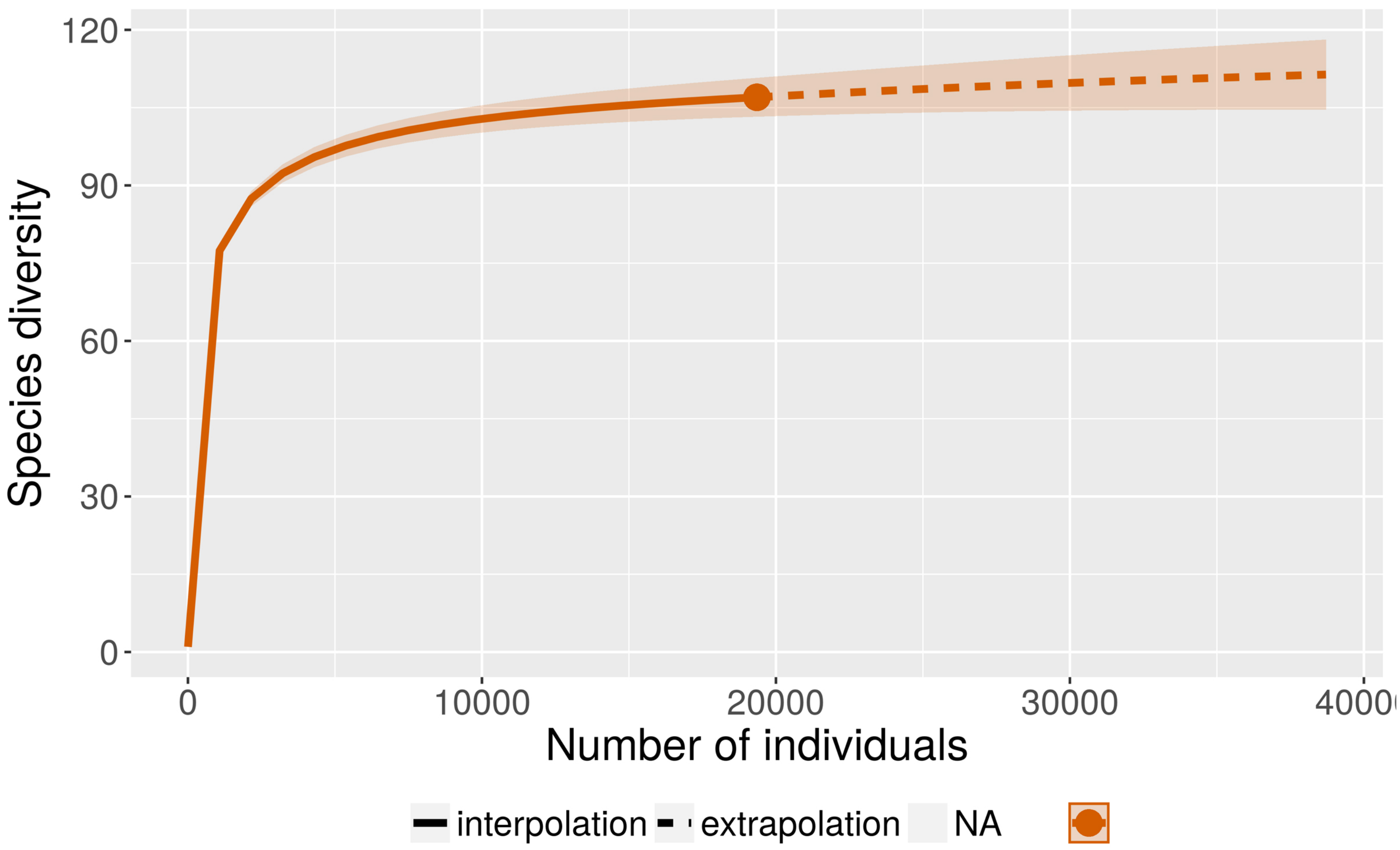

S1Fig Species accumulation curves for fish at each sampling section in the middle and lower reach of the Ganjiang River. Shaded areas represent the 95% confidence intervals.
