# Supplementary material for: Fish diversity in the middle and lower reaches of the Ganjiang River of China: Threats and conservation
Source: PLoS One. 2018 Nov 2;13(11):e0205116. doi: 10.1371/journal.pone.0205116 (PMC6214499; doi:10.1371/journal.pone.0205116)
Supplement: S1 Table — (DOCX) [file pone.0205116.s002.docx]

**S1Table** Habitat characteristics of the seven sampling sections in the middle and lower reaches of the Ganjiang River

| Sampling section | Latitude | Longitude | Altitude (m) | Water depth (m) | Width (m) | Habitat descriptions |
| --- | --- | --- | --- | --- | --- | --- |
| Nanchang section | 28°32'16″-28°44'09″ | 115°48'54″-115°53'38″ | 22-25 | 6.8-9.2 | 800-1300 | sparse vegetation, riverbed with sandstone and silt, rapid water flow |
| Baqiu section | 27°33′04″-27°37'37″ | 115°09′44″-115°14'09″ | 33-35 | 3.1-5.5 | 400-500 | abundant vegetation, riverbed with sandstone, rock and gravel, rapid water flow, |
| Jiangsha section | 27°30′53″-27°32'01″ | 115°07′41″-115°08′49″ | 38-40 | 4.7-16.9 | 600-1000 | sparse vegetation, riverbed with sandstone, rapid water flow |
| Wanhe section | 26°54′38″-26°57'36″ | 114°58′59″-115°15'36″ | 49-50 | 1.3-7.1 | 700-900 | sparse vegetation, riverbed with sandy substrate, slow water flow |
| Yanxi section | 26°46′19″-26°51'39″ | 114°54′10″-115°01'30″ | 53-55 | 4.8-13.0 | 700-800 | abundant vegetation, riverbed with sandstone and gravel, slow water flow |
| Shukou section | 26°44′56″-26°32'12″ | 114°49′54″-114°53'36″ | 60-63 | 2.3-8.0 | 700-1000 | abundant vegetation, riverbed with sandstone, slow water flow |
| Suichuan section | 26°08'01″-26°45′11″ | 114°07′53″-27°44'02″ | 328-617 | 1.0-3.0 | 50-200 | abundant vegetation, riverbed with sandstone, rock and gravel,  shallow water, rapid water flow, mountain streams |
