# Supplementary material for: Fish diversity in the middle and lower reaches of the Ganjiang River of China: Threats and conservation
Source: PLoS One. 2018 Nov 2;13(11):e0205116. doi: 10.1371/journal.pone.0205116 (PMC6214499; doi:10.1371/journal.pone.0205116)
Supplement: S3 Table — (DOCX) [file pone.0205116.s004.docx]

**S3Table.** Comparison of fish composition by sample period and sampling section in the middle and lower reaches of the Ganjiang River

| Species | Wet season (*IRI*%) | | | Dry season (*IRI*%) | | |
| --- | --- | --- | --- | --- | --- | --- |
|  | Channel | Reservoir | Tributary | Channel | Reservoir | Tributa  ry |
| *Coilia nasus* | 0.002 | 0.000 | 0.000 | 0.000 | 0.000 | 0.000 |
| *Coilia brachygnathus* | 0.089 | 0.000 | 0.000 | 0.000 | 0.000 | 0.000 |
| *Myxocyprinus asiaticus* | 0.001 | 0.085 | 0.080 | 0.008 | 0.000 | 0.000 |
| *Zacco platypus* | 0.000 | 1.134 | 6.750 | 0.000 | 0.000 | 3.006 |
| *Opsariichthys bidens* | 0.059 | 0.000 | 2.430 | 0.005 | 0.000 | 0.196 |
| *Mylopharyngodon piceus* | 4.320 | 0.510 | 0.000 | 1.559 | 0.161 | 0.000 |
| *Ctenopharyngodon idellus* | 11.494 | 2.830 | 6.970 | 3.145 | 0.273 | 3.053 |
| *Squaliobarbus curriculus* | 4.375 | 0.280 | 3.380 | 4.676 | 0.039 | 3.135 |
| *Ochetobius elongatus* | 0.001 | 0.000 | 0.000 | 0.003 | 0.000 | 0.000 |
| *Elopichthys bambusa* | 1.438 | 0.839 | 0.000 | 0.405 | 0.000 | 0.000 |
| *Sinibrama macrops* | 0.079 | 0.082 | 1.260 | 0.000 | 0.148 | 0.079 |
| *Sinibrama wui* | 0.000 | 0.009 | 0.000 | 0.000 | 0.000 | 0.000 |
| *Pseudolaubuca sinensis* | 0.000 | 0.000 | 0.940 | 0.000 | 0.028 | 0.102 |
| *Toxabramis swinhonis* | 0.001 | 0.000 | 0.000 | 0.000 | 0.000 | 0.000 |
| *Hemiculter leucisculus* | 0.927 | 2.622 | 3.450 | 0.726 | 1.668 | 1.022 |
| *Hemiculter bleekeri* | 5.267 | 1.440 | 0.300 | 0.000 | 0.000 | 1.001 |
| *Hemiculterella wui* | 0.000 | 0.000 | 0.600 | 0.000 | 0.000 | 0.165 |
| *Pseudohemiculter dispar* | 0.006 | 0.194 | 25.160 | 0.000 | 0.000 | 14.467 |
| *Chanodichthys erythropterus* | 0.111 | 0.000 | 0.000 | 0.000 | 0.000 | 0.000 |
| *Culter alburnus* | 5.362 | 2.058 | 0.000 | 6.885 | 0.092 | 0.000 |
| *Chanodichthys mongolicus* | 1.605 | 2.100 | 0.000 | 0.521 | 1.903 | 0.000 |
| *Chanodichthys dabryi* | 1.546 | 2.100 | 0.000 | 3.177 | 0.000 | 0.000 |
| *Parabramis pekinensis* | 0.940 | 0.176 | 0.250 | 1.367 | 4.311 | 0.123 |
| *Megalobrama terminalis* | 0.458 | 0.000 | 0.000 | 0.478 | 0.090 | 0.000 |
| *Megalobrama amblycephala* | 0.209 | 2.085 | 0.190 | 0.000 | 0.023 | 0.021 |
| *Xenocypris macrolepis* | 0.172 | 2.516 | 0.490 | 0.027 | 2.211 | 0.095 |
| *Xenocypris davidi* | 0.109 | 0.000 | 0.000 | 0.002 | 0.000 | 0.000 |
| *Distoechodon tumirostris* | 0.012 | 0.000 | 0.000 | 0.006 | 0.000 | 0.000 |
| *Pseudobrama simoni* | 3.682 | 0.000 | 0.000 | 2.144 | 0.000 | 0.000 |
| *Hypophthalmichthys molitrix* | 10.974 | 2.265 | 1.930 | 6.469 | 2.253 | 0.227 |
| *Hypophthalmichthys nobilis* | 2.313 | 0.162 | 0.460 | 1.518 | 0.389 | 0.097 |
| *Hemibarbus labeo* | 0.000 | 1.207 | 3.780 | 0.087 | 0.000 | 1.249 |
| *Hemibarbus maculatus* | 0.556 | 4.089 | 0.850 | 0.010 | 0.033 | 0.239 |
| *Pseudorasbora parva* | 0.028 | 0.365 | 0.000 | 0.000 | 1.509 | 0.000 |
| *Sarcocheilichthys sinensis* | 0.197 | 0.073 | 0.010 | 0.012 | 0.030 | 0.009 |
| *Sarcocheilichthys nigripinnis* | 0.001 | 0.412 | 0.000 | 0.000 | 0.000 | 0.000 |
| *Sarcocheilichthys kiangsiensis* | 0.154 | 0.522 | 0.000 | 0.001 | 0.184 | 0.000 |
| *Squalidus argentatus* | 7.750 | 26.152 | 18.290 | 8.023 | 12.631 | 13.879 |
| *Rhinogobio typus* | 0.071 | 0.053 | 0.000 | 0.011 | 0.009 | 0.000 |
| *Platysmacheilus exiguus* | 0.098 | 0.000 | 0.890 | 0.072 | 0.000 | 0.069 |
| *Huigobio chenhsienensis* | 0.000 | 0.000 | 0.130 | 0.000 | 0.000 | 0.037 |
| *Abbottina rivularis* | 0.429 | 0.000 | 0.080 | 0.003 | 0.000 | 0.141 |
| *Microphysogobio kiatingensis* | 0.004 | 0.000 | 0.100 | 0.000 | 0.000 | 0.010 |
| *Microphysogobio fukiensis* | 0.001 | 0.476 | 0.000 | 0.000 | 0.000 | 0.000 |
| *Microphysogobio elongatus* | 0.213 | 0.000 | 0.000 | 0.000 | 0.000 | 0.000 |
| *Pseudogobio guilinensis* | 0.000 | 0.000 | 1.600 | 0.000 | 0.000 | 0.017 |
| *Pseudogobio vaillanti* | 0.059 | 0.000 | 0.000 | 0.000 | 0.119 | 0.000 |
| *Saurogobio dabryi* | 0.546 | 11.116 | 1.590 | 1.301 | 9.256 | 0.077 |
| *Saurogobio xiangjiangensis* | 0.000 | 0.000 | 1.590 | 0.000 | 0.000 | 0.112 |
| *Gobiobotia tungi* | 0.000 | 0.008 | 0.000 | 0.000 | 0.000 | 0.000 |
| *Gobiobotia filifer* | 0.891 | 0.571 | 0.570 | 0.026 | 0.000 | 0.055 |
| *Acheilognathus macropterus* | 0.001 | 0.000 | 0.820 | 0.000 | 0.000 | 0.055 |
| *Acanthorhodeus chankaensis* | 0.001 | 0.062 | 0.000 | 0.000 | 0.000 | 0.000 |
| *Acheilognathus tonkinensis* | 0.012 | 0.000 | 0.000 | 0.000 | 0.000 | 0.000 |
| *Acheilognathus gracilis* | 0.000 | 0.015 | 0.260 | 0.002 | 0.000 | 0.008 |
| *Rhodeus ocellatus* | 0.016 | 0.000 | 0.030 | 0.000 | 0.000 | 0.021 |
| *Rhodeus lighti* | 0.041 | 0.000 | 0.000 | 0.000 | 0.000 | 0.000 |
| *Spinibarbus hollandi* | 0.000 | 0.000 | 0.670 | 0.000 | 0.000 | 0.314 |
| *Acrossocheilus paradoxus* | 0.000 | 0.000 | 0.320 | 0.000 | 0.000 | 0.070 |
| *Acrossocheilus parallens* | 0.001 | 0.000 | 7.670 | 0.000 | 0.000 | 4.035 |
| *Garra orientalis* | 0.000 | 0.000 | 2.560 | 0.000 | 0.000 | 0.010 |
| *Cyprinus carpio* | 12.735 | 13.406 | 0.340 | 14.622 | 10.980 | 1.036 |
| *Carassius auratus* | 9.809 | 8.495 | 0.360 | 7.888 | 9.066 | 0.945 |
| *Lepturichthys fimbriata* | 0.001 | 0.000 | 0.050 | 0.000 | 0.000 | 0.079 |
| *Erromyzon sinensis* | 0.000 | 0.000 | 0.030 | 0.000 | 0.000 | 0.007 |
| *Vanmanenia stenosoma* | 0.000 | 0.000 | 0.030 | 0.000 | 0.000 | 0.096 |
| *Cobitis sinensis* | 0.183 | 0.000 | 0.820 | 0.000 | 0.000 | 0.013 |
| *Misgurnus anguillicaudatus* | 0.089 | 1.189 | 0.150 | 0.103 | 1.182 | 0.179 |
| *Paramisgurnus dabryanus* | 0.014 | 0.568 | 2.010 | 0.000 | 0.000 | 0.031 |
| *Leptobotia elongata* | 0.000 | 0.000 | 12.880 | 0.000 | 0.000 | 5.728 |
| *Parabotia fasciata* | 0.124 | 0.544 | 1.520 | 0.000 | 0.000 | 0.022 |
| *Parabotia maculosa* | 0.011 | 0.034 | 0.000 | 0.000 | 0.000 | 0.000 |
| *Parabotia kiangsiensis* | 0.005 | 0.025 | 0.000 | 0.000 | 0.000 | 0.000 |
| *Parabotia banarescui* | 0.007 | 0.037 | 0.190 | 0.000 | 0.000 | 0.087 |
| *Tachysurus nitidus* | 1.594 | 0.383 | 0.000 | 2.018 | 0.018 | 0.000 |
| *Tachysurus fulvidraco* | 5.779 | 2.343 | 6.690 | 7.769 | 1.992 | 1.953 |
| *Pseudobagrus vachellii* | 0.309 | 0.000 | 0.000 | 0.000 | 0.095 | 0.000 |
| *Pelteobagrus eupogon* | 0.914 | 0.000 | 0.000 | 0.000 | 0.000 | 0.000 |
| *Pseudobagrus ondan* | 0.007 | 0.000 | 0.000 | 0.000 | 0.000 | 0.000 |
| *Pseudobagrus tenuis* | 0.045 | 0.078 | 0.370 | 0.000 | 0.000 | 0.048 |
| *Pseudobagrus pratti* | 0.000 | 0.000 | 0.090 | 0.000 | 0.000 | 0.052 |
| *Pseudobagrus crassilabris* | 0.031 | 0.028 | 0.910 | 0.024 | 0.081 | 0.056 |
| *Tachysurus dumerili* | 0.001 | 0.000 | 0.000 | 0.000 | 0.000 | 0.000 |
| *Hemibagrus macropterus* | 0.005 | 0.591 | 4.840 | 0.020 | 0.037 | 2.160 |
| *Silurus asotus* | 11.905 | 8.130 | 15.530 | 9.464 | 9.135 | 6.111 |
| *Silurus meridionalis* | 0.238 | 0.000 | 1.210 | 0.000 | 0.000 | 0.200 |
| *Pterocryptis cochinchinensis* | 0.000 | 0.000 | 0.320 | 0.000 | 0.000 | 0.188 |
| *Clarias fuscus* | 0.007 | 0.056 | 0.510 | 0.000 | 0.000 | 0.078 |
| *Liobagrus marginatus* | 0.001 | 0.000 | 0.000 | 0.000 | 0.000 | 0.000 |
| *Glyptothorax sinensis* | 0.000 | 0.000 | 0.100 | 0.000 | 0.000 | 0.012 |
| *Monopterus albus* | 0.191 | 0.000 | 5.540 | 0.091 | 0.000 | 1.311 |
| *Siniperca kneri* | 0.517 | 0.727 | 0.750 | 0.223 | 1.221 | 0.049 |
| *Siniperca chuatsi* | 4.736 | 1.211 | 0.730 | 3.447 | 1.836 | 0.043 |
| *Siniperca scherzeri* | 1.289 | 0.226 | 0.000 | 0.000 | 0.013 | 0.000 |
| *Siniperca obscura* | 0.000 | 0.000 | 0.050 | 0.000 | 0.000 | 0.010 |
| *Siniperca roulei* | 0.442 | 0.095 | 0.430 | 0.003 | 0.025 | 0.031 |
| *Odontobutis sinensis* | 0.125 | 0.337 | 0.000 | 0.008 | 0.000 | 0.000 |
| *Micropercops swinhonis* | 0.001 | 0.000 | 0.000 | 0.000 | 0.000 | 0.000 |
| *Rhinogobius giurinus* | 1.521 | 0.300 | 2.270 | 0.000 | 0.000 | 0.410 |
| *Rhinogobius cliffordpopei* | 0.109 | 0.015 | 0.000 | 0.000 | 0.000 | 0.000 |
| *Channa maculata* | 0.243 | 0.000 | 0.430 | 0.000 | 0.000 | 0.076 |
| *Channa asiatica* | 0.002 | 0.033 | 0.320 | 0.002 | 0.000 | 0.124 |
| *Channa argus* | 1.353 | 0.174 | 0.590 | 0.064 | 0.020 | 0.069 |
| *Macropodus opercularis* | 0.000 | 0.000 | 0.060 | 0.000 | 0.000 | 0.003 |
| *Macrognathus aculeatus* | 0.003 | 0.000 | 0.000 | 0.000 | 0.000 | 0.000 |
| *Sinobdella sinensis* | 0.000 | 0.000 | 0.370 | 0.000 | 0.000 | 0.001 |
| *Hyporhamphus intermedius* | 0.000 | 0.008 | 0.000 | 0.000 | 0.000 | 0.000 |
