# Supplementary material for: Fish diversity in the middle and lower reaches of the Ganjiang River of China: Threats and conservation
Source: PLoS One. 2018 Nov 2;13(11):e0205116. doi: 10.1371/journal.pone.0205116 (PMC6214499; doi:10.1371/journal.pone.0205116)
Supplement: S4 Table — Eu: Eurytopic; R: Rheophilic; L: Limnophilic; D: Diadromous; C: Piscivores; Zb: Zoobenthivores, I: Insectivores; O: Omnivores; Z: Zooplanktivores; P: Phytoplanktivores; Pb: Phytobenthivores; Herbivores; UL: Upper layer; LL: lower layer; De: demersal. △: Chinese endemic species. (DOCX) [file pone.0205116.s005.docx]

**S4Table.** Fish ecotype and Chinese Red List [37] in the middle and lower reaches of the Ganjiang River. Eu: Eurytopic; R: Rheophilic; L: Limnophilic; D: Diadromous; C: Piscivores; Zb: Zoobenthivores, I: Insectivores; O: Omnivores; Z: Zooplanktivores; P: Phytoplanktivores; Pb: Phytobenthivores; Herbivores; UL: Upper layer; LL: lower layer; De: demersal. △: Chinese endemic species.

| Species | Code | Life habits | Habitat characteristics | Feeding habits | Chinese Red List | |
| --- | --- | --- | --- | --- | --- | --- |
| *Coilia nasus* | Cnas | D | UL | C | LC | |
| *Coilia brachygnathus* | Cbra | Eu | UL | I | DD | |
| *Myxocyprinus asiaticus*△ | Masi | R | LL | I, Pb | CR | |
| *Zacco platypus* | Zpla | R | UL | Z, I | LC | |
| *Opsariichthys bidens* | Obid | R | UL | Z, I | LC | |
| *Mylopharyngodon piceus* | Mpic | D | LL | Zb | LC | |
| *Ctenopharyngodon idellus* | Cide | D | LL | H | LC | |
| *Squaliobarbus curriculus* | Scur | D | LL | O | LC | |
| *Ochetobius elongatus* | Oelo | D | UL | I | CR | |
| *Elopichthys bambusa* | Ebam | Eu | LL | C | LC | |
| *Sinibrama macrops*△ | Smac | R | LL | Z, I | LC | |
| *Sinibrama wui* | Swui | R | LL | O | LC | |
| *Pseudolaubuca sinensis* | Psin | Eu | UL | Z, I | LC | |
| *Toxabramis swinhonis*△ | Tswi | R | UL | Z | LC | |
| *Hemiculter leucisculus* | Hleu | L | UL | O | LC | |
| *Hemiculter bleekeri* | Hble | L | UL | O | LC | |
| *Hemiculterella wui* △ | Hwui | L | UL | O | LC | |
| *Pseudohemiculter dispar* | Pdis | L | UL | I | LC | |
| *Chanodichthys erythropterus* | Cery | L | UL | C | LC | |
| *Culter alburnus* | Calb | L | UL | C | LC | |
| *Chanodichthys mongolicus* | Cmon | L | UL | C | LC | |
| *Chanodichthys dabryi* | Cdab | L | UL | C | LC | |
| *Parabramis pekinensis* | Ppek | Eu | LL | H | LC | |
| *Megalobrama terminalis* | MTar | L | LL | H | LC | |
| *Megalobrama amblycephala*△ | Mamb | L | LL | H | LC | |
| *Xenocypris macrolepis* | Xarg | Eu | LL | Pb | DD | |
| *Xenocypris davidi* △ | Xdav | Eu | LL | Pb | LC | |
| *Distoechodon tumirostris*△ | Dtum | Eu | UL | Pb | LC | |
| *Pseudobrama simoni*△ | Psim | Eu | LL | Pb | LC | |
| *Hypophthalmichthys molitrix* | Hmol | D | UL | P | LC | |
| *Hypophthalmichthys nobilis* | Anob | D | UL | Z | LC | |
| *Hemibarbus labeo* | Hlab | R | LL | Zb | LC | |
| *Hemibarbus maculatus* | Hmac | Eu | LL | Zb, I | LC | |
| *Pseudorasbora parva* | Ppar | Eu | UL | I | LC | |
| *Sarcocheilichthys sinensis* | Ssin | Eu | LL | Zb, Pb | LC | |
| *Sarcocheilichthys nigripinnis*△ | Snig | Eu | LL | Zb, Pb | LC | |
| *Sarcocheilichthys kiangsiensis*△ | Skia | Eu | LL | Pb | LC | |
| *Squalidus argentatus* | Sarg | Eu | LL | I | LC | |
| *Rhinogobio typus*△ | Rtyp | R | De | Zb | LC | |
| *Platysmacheilus exiguus* | Pexi | R | De | Zb | LC | |
| *Huigobio chenhsienensis*△ | Hche | R | LL | O | LC | |
| *Abbottina rivularis* | Ariv | Eu | De | I | LC | |
| *Microphysogobio kiatingensis*△ | Mkia | R | De | Zb | DD | |
| *Microphysogobio fukiensis*△ | Mfuk | R | De | Zb | DD | |
| *Microphysogobio elongatus*△ | Melo | R | De | Zb | DD | |
| *Pseudogobio guilinensis* | Pgui | R | De | O | LC | |
| *Pseudogobio vaillanti* | Pvai | R | De | I | LC | |
| *Saurogobio dabryi* | Sdab | Eu | De | I | LC | |
| *Saurogobio xiangjiangensis*△ | Sxia | R | De | O | LC | |
| *Gobiobotia tungi*△ | GFan | R | De | P, Zb | DD | |
| *Gobiobotia filifer*△ | Gfil | R | De | Zb | LC | |
| *Acheilognathus macropterus* | Amac | Eu | LL | Pb | LC | |
| *Acanthorhodeus chankaensis* | Acha | Eu | LL | Pb | LC | |
| *Acheilognathus tonkinensis* | Aton | Eu | LL | Pb | LC | |
| *Acheilognathus gracilis*△ | Agra | Eu | LL | Pb | LC | |
| *Rhodeus ocellatus* | Roce | Eu | LL | Pb | LC | |
| *Rhodeus lighti* | Rlig | Eu | LL | Z, I | DD | |
| *Spinibarbus hollandi* | Shol | R | LL | O | LC | |
| *Acrossocheilus paradoxus* | Alab | R | De | O | DD | |
| *Acrossocheilus parallens*△ | Apar | R | LL | Pb, I | LC | |
| *Garra orientalis* | Gori | R | De | Pb | LC | |
| *Cyprinus carpio* | Ccar | Eu | LL | O | LC | |
| *Carassius auratus* | Caur | Eu | LL | O | LC | |
| *Lepturichthys fimbriata*△ | Lfim | R | De | Pb | DD | |
| *Erromyzon sinensis*△ | Psin | R | De | O | DD | |
| *Vanmanenia stenosoma*△ | Vste | R | De | Pb | DD | |
| *Cobitis sinensis* | Csin | R | De | I | LC | |
| *Misgurnus anguillicaudatus* | Mang | Eu | De | I | LC | |
| *Paramisgurnus dabryanus* | Pdab | Eu | De | I | LC | |
| *Leptobotia elongata*△ | Lelo | R | De | I | VU | |
| *Parabotia fasciata*△ | Pfas | R | De | I | LC | |
| *Parabotia maculosa*△ | Pmac | R | De | I | LC | |
| *Parabotia kiangsiensis*△ | Pkia | R | De | I | DD | |
| *Parabotia banarescui*△ | Pban | R | De | C | LC | |
| *Tachysurus nitidus*△ | Pnit | Eu | De | C, I | LC | |
| *Tachysurus fulvidraco* | Pful | Eu | De | C, I | LC | |
| *Pseudobagrus vachellii* | Pvac | Eu | De | I | LC | |
| *Pelteobagrus eupogon*△ | Peup | Eu | De | C, I | LC | |
| *Pseudobagrus ondan*△ | Pond | R | De | C, I | DD | |
| Pseudobagrus tenuis△ | Pten | R | De | C, I | DD | |
| *Pseudobagrus pratti*△ | Ppra | R | De | O | VU | |
| *Pseudobagrus crassilabris*△ | Lcra | R | De | O | LC | |
| *Tachysurus dumerili* | Llon | R | De | C, I | LC | |
| *Hemibagrus macropterus*△ | Mmac | R | De | C, I | LC | |
| *Silurus asotus* | Saso | Eu | De | C | LC | |
| *Silurus meridionalis*△ | Ssol | Eu | De | C | LC | |
| *Pterocryptis cochinchinensis* | Scoc | Eu | LL | C | LC |  |
| *Clarias fuscus* | Cfus | Eu | De | C | LC | |
| *Liobagrus marginatus*△ | Lmar | R | De | I | DD | |
| *Glyptothorax sinensis*△ | Gsin | R | De | I, Pb | LC | |
| *Monopterus albus* | Malb | Eu | De | I | LC | |
| *Siniperca kneri* | Skne | Eu | LL | C | LC | |
| *Siniperca chuatsi* | Schu | Eu | LL | C | LC | |
| *Siniperca scherzeri* | Ssch | Eu | LL | C | LC | |
| *Siniperca obscura*△ | Sobs | Eu | UL | C | NT | |
| *Siniperca roulei*△ | Srou | Eu | LL | C | VU | |
| *Odontobutis sinensis*△ | Osin | Eu | De | C | LC | |
| *Micropercops swinhonis* | Mswi | Eu | De | Zb | LC | |
| *Rhinogobius giurinus* | Rgiu | Eu | De | I | LC | |
| *Rhinogobius cliffordpopei*△ | Rcli | Eu | De | Zb | LC | |
| *Channa maculata* | Cmac | Eu | De | C | LC | |
| *Channa asiatica* | Casi | Eu | De | C | LC | |
| *Channa argus* | Carg | Eu | De | C | LC | |
| *Macropodus opercularis* | Mope | R | LL | O | LC | |
| *Macrognathus aculeatus*△ | Macu | Eu | De | C, I | LC | |
| *Sinobdella sinensis* | Msin | R | De | C | DD | |
| *Hyporhamphus intermedius* | Hint | Eu | UL | Z | LC | |
