# Supplementary material for: Fish diversity in the middle and lower reaches of the Ganjiang River of China: Threats and conservation
Source: PLoS One. 2018 Nov 2;13(11):e0205116. doi: 10.1371/journal.pone.0205116 (PMC6214499; doi:10.1371/journal.pone.0205116)
Supplement: S5 Table — (DOCX) [file pone.0205116.s006.docx]

**S5Table.** Historical fish composition and distribution in the middle and lower reaches of the Ganjiang River

|  | 1982~1991 | 2007~2010 |
| --- | --- | --- |
| **Acipenseridae** |  |  |
| *Acipenser sinensis* Gray, 1835 | + |  |
| **Clupeidae** |  |  |
| *Tenualosa reevesii*(Richardson, 1846) | + |  |
| **Engraulidae** |  |  |
| *Coilia nasus* Temminck & Schlegel, 1846 | + |  |
| *Coilia brachygnathus* Kreyenberg & Pappenheim, 1908 | + | + |
| **Salangidae** |  |  |
| *Protosalanx hyalocranius* (Abbott, 1901) | + |  |
| *Hemisalanx brachyrostralis* (Fang, 1934) | + |  |
| **Catostomidae** |  |  |
| *Myxocyprinus asiaticus* (Bleeker, 1864) |  |  |
| **Cyprinidae** |  |  |
| *Zacco platypus* (Temminck et Schlegel, 1846) | + | + |
| *Opsariichthys bidens* (Günther, 1873) | + | + |
| *Aphyocypris chinensis* (Günther, 1868) | + |  |
| *Rhynchocypris oxycephalus* (Sauvage & Dabry, 1874) | + |  |
| *Rhynchocypris lagowskii* (Dybowski, 1869) | + |  |
| *Mylopharyngodon piceus* (Richardson, 1846) | + | + |
| *Ctenopharyngodon idellus* (Valenciennes, 1844) | + | + |
| *Squaliobarbus curriculus* (Richardson, 1846) | + | + |
| *Ochetobius elongatus* (Kner,1867) | + | + |
| *Luciobrama macrocephalus*(Lacepède, 1803) | + |  |
| *Elopichthys bambusa* (Richardson, 1845) | + | + |
| *Sinibrama macrops* (Günther, 1868) | + | + |
| *Sinibrama wui*(Rendahl, 1933) | + |  |
| *Ancherythroculter kurematsui*(Kimura, 1934) |  | + |
| *Pseudolaubuca sinensis* Bleeker, 1864 | + | + |
| *Pseudolaubuca engraulis*(Nichols, 1925) | + | + |
| *Toxabramis swinhonis* Günther, 1873 | + | + |
| *Hemiculter leucisculus*(Basilewsky, 1855) | + | + |
| *Hemiculter bleekeri* Warpachowsky, 1888 | + | + |
| *Hemiculterella sauvagei*Warpachowski, 1888 | + |  |
| *Hemiculterella wui* (Wang, 1935) |  | + |
| *Pseudohemiculter dispar*(Peters, 1881) | + | + |
| *Pseudohemiculter hainanensis*(Boulenger, 1900) | + |  |
| *Chanodichthys erythropterus* (Basilewsky, 1855) | + | + |
| *Culter alburnus* (Basilewsky) | + | + |
| *Chanodichthys mongolicus* (Basilesky, 1855) | + | + |
| *Chanodichthys oxycephalus* (Bleeker, 1871) | + | + |
| *Culter oxycephaloides*Kreyenberg & Pappenheim, 1908 | + |  |
| *Chanodichthys dabryi* (Bleeker, 1871) | + | + |
| *Parabramis pekinensis* (Basilesky, 1855) | + | + |
| *Megalobrama terminalis* (Richardson, 1846) | + | + |
| *Megalobrama amblycephala* Yih, 1955 | + | + |
| *Xenocypris macrolepis* Bleeker, 1871 | + | + |
| *Xenocypris davidi* Bleeker, 1871 | + | + |
| *Plagiognathops microlepis* (Bleeker, 1871) | + | + |
| *Distoechodon tumirostris* Peters, 1881 | + | + |
| *Pseudobrama simoni* (Bleeker, 1864) | + | + |
| *Hypophthalmichthys molitrix* (Valenciennes, 1844) | + | + |
| *Hypophthalmichthys nobilis* (Richardson, 1845) | + | + |
| *Hemibarbus labeo* (Pallas, 1776) | + | + |
| *Hemibarbus longirostris* (Regan, 1908) | + |  |
| *Hemibarbus maculatus* Bleeker, 1871 | + | + |
| *Hemibarbus umbrifer* (Lin, 1931) | + |  |
| *Paracanthobrama guichenoti* Bleeker, 1864 | + |  |
| *Belligobio nummifer*(Boulenger, 1901) |  | + |
| *Pseudorasbora parva* (Temminck & Schlegel, 1846) | + | + |
| *Sarcocheilichthys sinensis* Bleeker, 1871 | + | + |
| *Sarcocheilichthys parvus* Nichols, 1930 | + | + |
| *Sarcocheilichthys nigripinnis* (Günther, 1873) | + | + |
| *Sarcocheilichthys kiangsiensis* Nichols, 1930 | + | + |
| *Gnathopogon polytaenia*(Nichols, 1925) | + |  |
| *Gnathopogon imberbis* (Sauvage *&*Dabry, 1874) |  | + |
| *Squalidus argentatus* (Sauvage & Dabry, 1874) | + | + |
| *Squalidus wolterstorffi*(Regan, 1908) | + |  |
| *Coreius heterodon* (Bleeker, 1864) | + | + |
| *Rhinogobio typus* Bleeker, 1871 | + | + |
| *Rhinogobio cylindricus* (Günther, 1888) | + |  |
| *Rhinogobio ventralis*Sauvage & Dabry, 1874 | + |  |
| *Platysmacheilus exiguus* (Lin, 1932) | + | + |
| *Platysmacheilus longibarbatus*Lu, Luo & Chen, 1977 | + |  |
| *Huigobio chenhsienensis* Fang, 1938 | + |  |
| *Abbottina rivularis* (Basilewsky, 1855) | + | + |
| *Abbottina obtusirostris* (Wu & Wang, 1931) | + |  |
| *Microphysogobio tungtingensis* (Nichols, 1926) | + |  |
| *Microphysogobio kiatingensis* (Wu, 1930) | + | + |
| *Microphysogobio fukiensis* (Nichols, 1926) | + | + |
| *Microphysogobio elongatus* (Yao & Yang, 1977) | + | + |
| *Pseudogobio vaillanti* (Sauvage, 1878) | + | + |
| *Pseudogobio guilinensis* Yao & Yang, 1977 | + |  |
| *Saurogobio dabryi* Bleeker, 1871 | + | + |
| *Saurogobio dumerili* Bleeker, 1871 | + |  |
| *Saurogobio xiangjiangensis* Tang, 1980 | + |  |
| *Saurogobio gymnocheilus* Lo, Yao & Chen, 1998 | + |  |
| *Saurogobio gracilicaudatus* Yao & Yang, 1977 | + |  |
| *Gobiobotia tungi* Fang, 1933 | + |  |
| *Gobiobotia filifer* (Garman, 1912) | + | + |
| *Gobiobotia longibarba*Fang & Wang, 1931 | + |  |
| *Gobiobotia meridionalis*Chen & Cao, 1977 | + |  |
| *Acheilognathus macropterus* (Bleeker, 1871) | + | + |
| *Acanthorhodeus chankaensis* (Dybowski, 1872) | + | + |
| *Acheilognathus tonkinensis* (Vaillant, 1892) | + | + |
| *Acheilognathus barbatulus* Günther, 1873 | + | + |
| *Acheilognathus gracilis* Nichols, 1926 | + | + |
| *Acheilognathus polylepis* (Wu, 1964) | + |  |
| *Acheilogiathus hypselonotus* (Bleeker, 1871) | + | + |
| *Acheilognathus imberbis* Günther, 1868 | + |  |
| *Tanakia himantegus* (Günther, 1868) | + | + |
| *Acheilognathus meridianus* (Wu, 1939) | + |  |
| *Rhodeus ocellatus* (Kner, 1866) | + | + |
| *Rhodeus lighti* (Wu, 1931) | + | + |
| *Spinibarbus hollandi* Oshima, 1919 | + | + |
| *Spinibarbus sinensis* (Bleeker, 1871) | + | + |
| *Barbodes semifasciolatus* (Günther,1868) | + |  |
| *Acrossocheilus fasciatus*(Steindachner, 1892) | + |  |
| *Acrossocheilus paradoxus* (Günther, 1868) | + |  |
| *Acrossocheilus parallens* (Nichols, 1931) | + | + |
| *Acrossocheilus hemispinus*(Nichols, 1925) | + |  |
| *Acrossocheilus kreyenbergii* (Regan, 1908) | + |  |
| *Acrossocheilus elongates* (Pellegrin et Chevey) | + |  |
| *Onychostoma barbatulum* (Pellegrin, 1908) | + |  |
| *Onychostoma simum* (Sauvage & Dabry, 1874) | + |  |
| *Onychostoma lini*(Wu, 1939) | + |  |
| *Folifer brevifilis* (Peters, 1881) | + |  |
| *Pseudogyrincheilus procheilus* (Sauvage & Dabry, 1874) | + |  |
| *Garra orientalis* Nichols, 1925 | + | + |
| *Cyprinus carpio* Linnaeus, 1758 | + | + |
| *Carassius auratus* (Linnaeus, 1758) | + | + |
| **Homalopteridae** |  |  |
| *Lepturichthys fimbriata* (Günther, 1888) | + | + |
| *Pseudogastromyzon changtingensis* Liang, 1942 | + |  |
| *Vanmanenia stenosoma* (Boulenger, 1901) | + |  |
| *Vanmanenia pinchowensis* (Fang, 1935) |  | + |
| *Vanmanenia xinyiensis* Zheng & Chen, 1980 |  | + |
| *Vanmanenia gymnetrus* Chen, 1980 | + |  |
| *Formosania stigmata* (Nichols, 1926) |  | + |
| **Cobitidae** |  |  |
| *Cobitis sinensis* (Sauvage et Dabry, 1874) | + | + |
| *Cobitis macrostigma*Dabry et Thiersant, 1872 | + |  |
| *Misgurnus anguillicaudatus* (Cantor, 1842) | + | + |
| *Paramisgurnus dabryanus* Dabry de Thiersant, 1872 | + | + |
| *Parabotia fasciata* Dabry de Thiersant, 1872 | + | + |
| *Parabotia kiangsiensis* Liu & Guo, 1986 |  | + |
| *Parabotia maculosa* (Wu, 1939) | + | + |
| *Parabotia banarescui* (Nalbant, 1965) | + | + |
| *Leptobotia taeniops* (Sauvage, 1878) | + | + |
| *Leptobotia elongata* (Bleeker, 1870) | + |  |
| *Leptobotia tientainensis* (Wu, 1930) | + |  |
| *Leptobotia tchangi* Fang, 1936 | + |  |
| *Nemacheilus singhi* Menon, 1987 | + |  |
| *Schistura fasciolata* (Nichols & Pope, 1927) | + |  |
| *Schistura incerta* (Nichols, 1931) | + |  |
| *Protonemacheilus longipectoralis* Yang & Chu, 1990 |  | + |
| **Bagridae** |  |  |
| *Tachysurus nitidus* (Sauvage & Dabry, 1874) | + | + |
| *Tachysurus fulvidraco* (Richardson, 1846) | + | + |
| *Pseudobagrus vachellii* (Richardson, 1846) | + | + |
| *Pelteobagrus eupogon* (Boulenger, 1892) | + | + |
| *Pseudobagrus ondan* Shaw, 1930 | + | + |
| *Pseudobagrus tenuis* (Günther, 1873) | + | + |
| *Pseudobagrus ussuriensis* (Dybowski,1872) | + |  |
| *Pseudobagrus truncatus* (Regan, 1913) | + |  |
| *Pseudobagrus pratti* (Günther, 1892) | + |  |
| *Pseudobagrus brericaudatus* (Wu, 1930) | + |  |
| *Pseudobagrus analis* (Nichols, 1930) | + |  |
| *Pseudobagrus taeniatus* (Günther, 1873) | + |  |
| *Leiocassis crassilabris* (Günther, 1864) | + | + |
| *Tachysurus dumerili* (Bleeker, 1864) | + |  |
| *Pseudobagrus albomarginatus* (Rendahl, 1928) | + |  |
| *Hemibagrus macropterus* Bleeker, 1870 | + | + |
| **Siluridae** |  |  |
| *Silurus asotus* Linnaeus, 1758 | + | + |
| *Pterocryptis cochinchinensis* (Valenciennes, 1840) | + |  |
| *Silurus meridionalis* Chen, 1977 | + | + |
| **Amblycipitidae** |  |  |
| *Liobagrus marginatus* (Günther, 1892) | + | + |
| *Liobagrus anguillicanda* Nichols, 1926 | + |  |
| **Sisoridae** |  |  |
| *Glyptothorax sinensis*(Regan, 1908) | + |  |
| *Glyptothorax fokiensis* (Rendahl, 1925) | + | + |
| **Clariidae** |  |  |
| *Clarias fuscus* (Lacepède, 1803) | + | + |
| **Synbranchidae** |  |  |
| *Monopterus albus*(Zuiew, 1793) | + | + |
| **Serranidae** |  |  |
| *Siniperca kneri* Garman, 1912 | + | + |
| *Siniperca chuatsi* (Basilewsky, 1855) | + | + |
| *Siniperca scherzeri* Steindachner, 1892 | + | + |
| *Siniperca obscura* Nichols, 1930 | + |  |
| *Siniperca undalata*Fang & Chong, 1932 | + |  |
| *Siniperca roulei* Wu, 1930 | + | + |
| **Odontobutidae** |  |  |
| *Odontobutis sinensis* Wu, Chen & Chong, 2002 | + | + |
| *Micropercops swinhonis*(Günther, 1873) | + | + |
| **Gobiidae** |  |  |
| *Rhinogobius giurinus* (Rutter, 1897) | + | + |
| *Rhinogobius cliffordpopei* (Nichols, 1925) | + | + |
| *Rhinogobius lindbergi* Berg, 1933 | + |  |
| *Rhinogobius leavelli* (Herre, 1935) | + |  |
| **Belontiidae** |  |  |
| *Macropodus opercularis* (Linnaeus, 1758) | + | + |
| **Channidae** |  |  |
| *Channa maculata* (Lacepède, 1801) | + | + |
| *Channa asiatica* (Linnaeus, 1758) | + |  |
| *Channa argus* (Cantor, 1842) | + | + |
| **Mastacembelidae** |  |  |
| *Macrognathus aculeatus* (Bloch, 1786) | + | + |
| *Mastacembelus armatus* (Lacepède, 1800) | + |  |
| **Hemiramphidae** |  |  |
| *Hyporhamphus intermedius* (Cantor, 1842) | + | + |
| **Anguillidae** |  |  |
| *Anguilla japonica* Temminck & Schlegel, 1846 | + | + |
| **Cyprinodontidae** |  |  |
| *Oryzias latipes* (Temminck & Schlegel, 1846) | + |  |
| **Cynoglossidae** |  |  |
| *Cynoglossus gracilis* Günther, 1873 | + |  |
| **Tetrodontidae** |  |  |
| *Takifugu ocellatus* (Linnaeus, 1758) | + |  |
| *Takifugu obscurus* (Abe, 1949) | + |  |
